# Network dynamics of the yeast methyltransferome

**DOI:** 10.15698/mic2019.08.687

**Published:** 2019-07-09

**Authors:** Guri Giaever, Elena Lissina, Corey Nislow

**Affiliations:** 1 Department of Pharmaceutical Sciences, University of British Columbia, Vancouver, BC, Canada V6T 1Z3.

**Keywords:** SAM methyltransferase, histone methyltransferase, genetic interaction, phospholipid, COMPASS

## Abstract

Sulfur assimilation and the biosynthesis of methionine, cysteine and S-adenosylmethionine (SAM) are critical to life. As a cofactor, SAM is required for the activity of most methyltransferases (MTases) and as such has broad impact on diverse cellular processes. Assigning function to MTases remains a challenge however, as many MTases are partially redundant, they often have multiple cellular roles and these activities can be condition-dependent. To address these challenges, we performed a systematic synthetic genetic analysis of all pairwise MTase double mutations in normal and stress conditions (16°C, 37°C, and LiCl) resulting in an unbiased comprehensive overview of the complexity and plasticity of the methyltransferome. Based on this network, we performed biochemical analysis of members of the histone H3K4 COMPASS complex and the phospholipid methyltransferase OPI3 to reveal a new role for a phospholipid methyltransferase in mediating histone methylation (H3K4) which underscores a potential link between lipid homeostasis and histone methylation. Our findings provide a valuable resource to study methyltransferase function, the dynamics of the methyltransferome, genetic crosstalk between biological processes and the dynamics of the methyltransferome in response to cellular stress.

## INTRODUCTION

S-adenosylmethionine (SAM) is a universal cofactor found in all branches of life (e.g. viruses, plants, bacteria, yeast and human) where it plays a critical role in the transfer of methyl groups to diverse biomolecules, including DNA, proteins and small-molecules. These substrates are involved in numerous biological processes including signal transduction, chromatin remodeling, gene regulation, DNA repair, and ageing [1]. Methyltransferase (MTase) enzymes transfer a methyl group from SAM to their substrates, forming S-adenosylhomocysteine (SAH) which is further metabolized to homocysteine, a precursor for methionine, cysteine and glutathione. Not unexpectedly, SAM dependent MTases are crucial for essential cellular functions and, when dysregulated, can cause disease. For example, recent work has shown that the DNA MTases, protein MTases and RNA MTases are directly involved in the epigenetic regulation of gene expression during cancer development and progression [2].

Compared to other proteins that transfer high energy metabolites such as kinases, phosphatases, and acetylases, MTases are unique in the diversity of their substrates and also in their ability to target multiple atoms (e.g. O, C, N, S and halides). This substrate flexibility, combined with the poor sequence conservation in their active site domains [3] complicates assignment of function based on primary sequence. Functional data is often useful for such characterization, but the fact that the majority of MTs are not essential for viability in laboratory conditions and may share overlapping function complicates their characterization. To address this challenge, we systematically generated MTase double mutants and analyzed their fitness in the absence and presence of environmental stress.

Although the majority of yeast MTases are at least partially characterized (i.e. at the primary or secondary sequence level), between 15-20% have no known function [4, 5]. While traditional, focused approaches are useful for understanding individual MTases, comprehensive perturbation strategies will likely be required to understand those MTases that are non-essential and which lack overt phenotypes due to potentially shared and compensatory mechanisms. Indeed, a model of one MTase-one substrate is an oversimplification that is not supported by experimental evidence, and which complicates efforts to uncover function. For example, the human Euchromatic histone-lysine N-MTase 2 (*EHMT2*) catalyzes a specific methylation of histone H3 at lysine residue 9, yet the same enzyme also methylates non-histone targets such as p53 [6, 7]. Similarly, in plants, the *OMT2* MTase modifies structurally diverse small molecules [8]. Consistent with their multiple roles, MTases often contain distinct functional domains; with at least half of all yeast MTases possessing both RNA-binding domains and lipid-binding motifs [5, 9].

Genome-scale screens of double deletions have proved powerful for tackling the inherent robustness of biological systems [10], in revealing phenotypes in partially redundant systems, and providing valuable insight into gene function, and genetic network architecture [11–14]. Furthermore, the addition of external perturbations [15] to such genetically challenged cells can provide additional insight into gene function. Synthetic Genetic Array (SGA) technology is a powerful tool for automating construction of double mutants [11, 12] to allow the fitness of each double mutant to be assessed by colony size measurements. In SGA, a double mutant exhibiting fitness defects greater than or less than the expected multiplicative effect of the combined fitness of each single mutant define negative and positive genetic interactions, respectively.

Here we quantify the genetic interactions in *Saccharomyces cerevisiae* between SAM MTases by constructing a reference set comprised of all possible pairwise double MTase mutant strains. We quantify the dynamics of these interactions in response to a variety of environmental stress conditions (16°C, 37°C, and 0.25 mM LiCl) selected from a survey of a broader list of environmental stress responses [16]. The resulting genetic interaction network represents the first comprehensive view of the methyltransferome and the dynamics in response to stress. Based on this network, biochemical analysis of a genetic relationship observed between members of the histone COMPASS complex and the phospholipid MT Opi3 revealed a novel role of phospholipid MTs in mediating histone methylation, indicating that these two distinct MTs cooperate to affect fundamental biological processes. We highlight our key findings and include examples of how the yeast methyltransferome network provides insight into MT function that can be leveraged in more focused studies in other biological systems, including human.

## RESULTS

### Construction of MTase double mutants and evaluation of their genetic interactions

**Figure 1:**
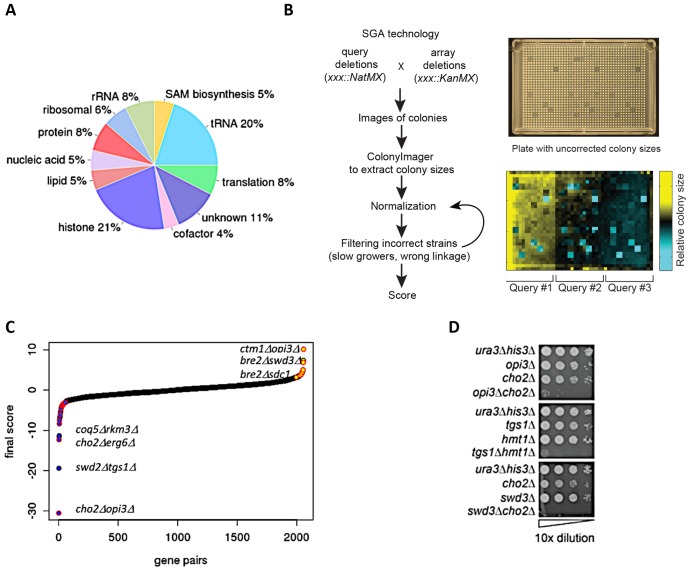
FIGURE 1: Construction of double MT mutants and evaluation of their genetic interactions. **(A)** Classification of methyltransferases by function/predicted substrate. **(B)** SGA procedure for double mutant generation and data processing. A representative image of a ‘final' plate used for the last step of selection of haploid double-deletion mutant colonies is shown. Each mutant is present in four replicates. Images are converted to pixels using Colony Imager software to obtain relative colony sizes. Each plate contains three query strains marked with NatR (*mtase*Δ*::NatMX*) crossed to an array of KanR-marked mutants (*mtase*Δ*::KanMX*). **(C)** Distribution of all MT genetic interactions; negative (blue), neutral (black) and positive (yellow); select double mutants outliers are labeled. Red outlines on each data point signify genetic interactions reported in previous studies [52]. **(D)** Validation of select negative genetic interactions by spot dilution growth assays, with strain genotypes indicated.

To systematically assess genetic interactions between AdoMet-dependent MTases in *S. cerevisiae*, we selected 94 MTase deletion mutants (known and putative) and four JmJ domain-containing demethylase mutants (*GIS1, RPH1, JHD1,* and *JHD2*). We also included eight essential RNA MTases as loss-of-function mutants (*ABD1, DIM1, GCD10, GCD14, NOP2, SPB1, SWD2, TRM5*) constructed as DAmP loss-of-function alleles [17, 18]. In total, our screen interrogated MTases involved in diverse biological processes and substrate specificity including: nucleic acids (tRNA, rRNA, mRNA, snRNA), proteins (histones, ribosomal proteins, transcription factors, etc.), small molecules (lipids, metabolites) and several with unknown substrates **(Figure 1A)**. To systematically assess genetic interactions between MTases in *S. cerevisiae*, we constructed all possible pairwise double mutants of the 94 MTases using SGA technology [11, 12, 14, 19, 20] **(Figure 1B)**. Genetic interactions were quantified by fitness of the MTase double mutant strains using colony size as a metric [21–23].

Crossing 81 Kan^R^-marked MTases with 94 Nat^R^-marked MTases resulted in a matrix comprised of 81 x 94 = 7614 reciprocal and (control) single genetic pairs **(Figure S1A)**. Consistent with previous observations, single mutant fitness defects have a large effect on each strain's genetic interaction profiles, and such ‘slow growing' stains exhibited a greater number of genetic interactions (e.g. *RSM22* and *OPI3*) compared to controls (e.g. *HIS3*) **(Figure S1B)** [12]. Because the mated strains in this matrix contain the selectable markers *NatMX* and *KanMX* in the exactly same chromosomal locations, one can use the resulting lethal phenotype of a double mutant (i.e. the strains on the diagonal in the matrix) in the presence of both selection drugs (G418 and nourseothricin) as a phenotype to check for mutant strain accuracy. To decrease the rate of false positive scores for any MTase genes located within 50 kb of each other, these gene pairs were also filtered out **(Figure S1C)** to avoid any possible genetic linkage (and failure to segregate during meiosis) that could confound our analysis. Because we found that the genetic profiles of the reciprocal strains (Nat^R^-Kan^R^ vs Kan^R^-Nat^R^) tend to correlate strongly to each other **(Figure S1D)**, we used the reciprocal fitness measures to identify and remove potentially incorrect strains prior to analysis.

To evaluate the reproducibility of our genetic interaction data, we compared the scores from two independent screens and found them highly similar (*r* =0.83) **(Figure S2A)**. When we restricted the comparison to only those scores deemed significant (based on the score threshold (|score|>2.5), the between-experiment correlation increased to *r* =0.92 (*p*-value <1x10^-200^). Because the majority of MTase double mutant strains (4032/5148) were constructed as independent (and reciprocal) double-deletion strains (Nat^R^-Kan^R^ and Kan^R^-Nat^R^), the scores for these reciprocal gene pairs were also compared. We found the correlation of *r =*0.68, *p-*value <1x10^-11^, |score| > 2.5 **(Figure S2B)**.

As a final benchmark, we estimated our false discovery rate by comparing the overlap of our genetic interactions with published large-scale SGA studies [11, 12] and found significant overlap (*r* =0.49, *p*-value <0.003) **(Figure S2C)**. After the removal of genetic interactions with opposite signs, we compiled a high confidence dataset of 2056 averaged scores (see Materials and Methods) **(Figure 1C)**. To evaluate the accuracy these genetic interaction scores, we tested a subset of significant negative and positive scores (threshold |score| >2.5) [22] using serial dilution spot assays **(Figure 1D, Figure S3)** and high resolution liquid growth assays **(Figure S4)**. In total, using visual inspection we estimated that the false positive rate for our screen was ~40% for negative genetic interactions and ~50% for positive genetic interactions.

Following the data processing and quality control steps described above (see also Materials and Methods) we obtained a final experimental matrix of 66 Kan^R^ x 78 Nat^R^-resistant MTase double deletion strains, representing 5148 double-mutants. Each row and column represents a genetic interaction profile for a particular MTase. In this representation, negative scores are seen to occur between genes in the same pathway or between those that share the same function, and conversely, positive scores are more likely to occur between physically interacting proteins in protein complexes. Based on our use of strict thresholds for data quality and reproducibility, we suggest that this systematic assessment of genetic interactions of yeast MTases and their resulting fitness will provide insight into the central role of SAM homeostasis in the cell.

### Genetic architecture of the yeast methyltransferome

**Figure 2:**
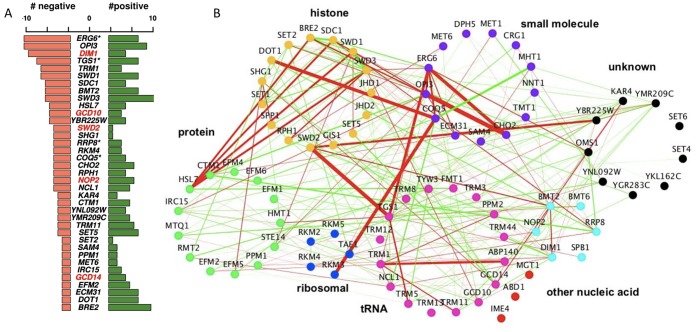
FIGURE 2: Genetic architecture of the yeast methyltransferome. **(A)** Frequency of high-confidence positive and negative genetic interactions (|score| >2.5) across the methyltransferome network. Essential genes are labeled in red; * indicates slow growers. **(B)** Genetic interaction network of the yeast methyltransferome under the standard growth condition (30°C). Each MT gene is represented as a node in the network and significant genetic interactions by edges. Nodes are colored according to the substrate type. Edge width represents the strength of the genetic, interaction score and edge color the interaction type (red negative, green positive).

The complete methyltransferome network comprises 2056 high-confidence interactions. Although the majority of genetic interactions were neutral (88.3%), the methyltransferome network was enriched for significant genetic interactions; ~11.8% (241 out of 2056, |score| >2.5) compared to the ~1-2% observed for randomly selected double deletion strains [11, 12]. This enrichment is consistent with other SGA studies that focus on a specific pathways or functionally related genes [17, 24, 25]. Interestingly, there is a prevalence of positive (alleviating) over negative (aggravating) genetic interactions in the dataset (~1.5x enrichment for ***positive*** interactions in contrast to the ~2x enrichment observed for ***negative*** interactions across the entire genome [11, 12]). MTases with greatest number of genetic interactions (i.e. those representing “hubs” in the network) included two MTases involved in lipid homeostasis (*ERG6* and *OPI3*), *DIM1,* an essential rRNA MT and *TGS1,* a snoRNA nucleolar MTase. As expected, MTases of unknown functions demonstrated the lowest number of genetic interactions, consistent with their dearth of their annotated phenotypes in the SGD (www.yeastgenome.org) **(Figure 2A)**.

We next assessed the methyltransferome genetic interaction network for correlation with their reported substrate specificity. The architecture of the MTase genetic interaction network, when visualized by substrate-accepting specificity (broadly classified as histone, protein, ribosomal, tRNA, rRNA, other nucleic acid, small molecules, and unknown) **(Figure 2B)** revealed genetic interactions (both positive and negative) both between and within the substrate-based clusters. In particular, we found the strong negative interactions connecting different substrate-acting MTases, suggesting that the functions for these MTases are not as categorical as their annotations might suggest. Rather, these unexpected between-cluster interactions likely reflect their capacities to buffer each other **(Figure 2B)**. For example, the nucleolar snRNA/snoRNA MTases *TGS1* exhibited strong negative interactions with the essential protein MTase *SWD2.* Consistent with this observation, Swd2 is known to be a subunit of the cleavage and polyadenylation factor complex and to play a role in snoRNA 3' end formation [26]. In another example, the arginine MTase *HSL7* showed strong negative genetic interactions with several members of the evolutionarily conserved histone H3K3 MTase COMPASS complex, including *SDC1, SWD3, SWD1,* and *BRE2*. This observation suggests a biological interaction between these crucial regulators of transcriptional regulation and the *HSL7* arginine MTase in cells deleted for these genes **(Figure 2B)**. Notably, the complementing human homolog of *HSL7, PRMT5* also supports a link to transcription [27, 28]. These observations suggest that the high interconnectedness of the yeast methyltransferome genetic network is not restricted by MTase-substrate relationships and that the MTase network displays unanticipated, between-cluster relationships.

**Figure 3:**
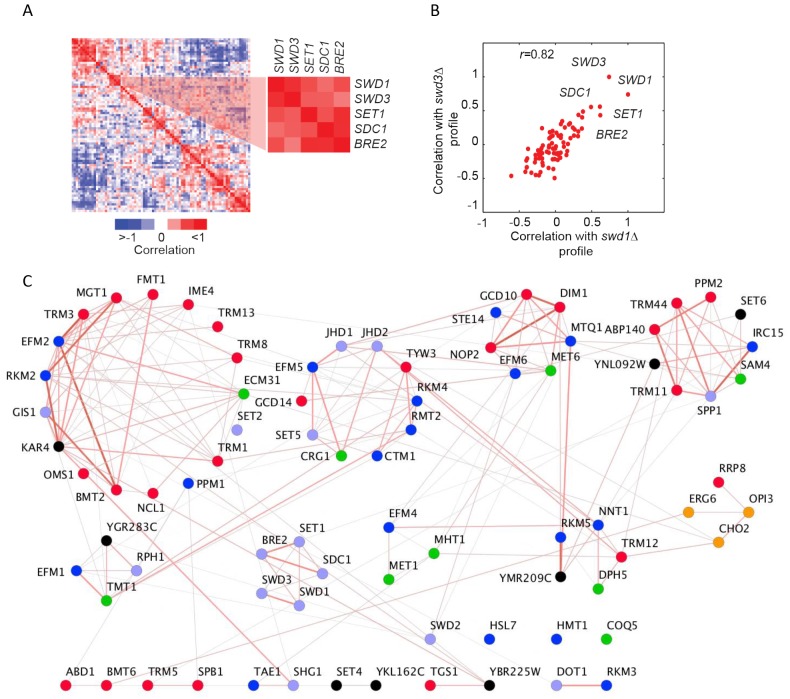
FIGURE 3: Genetic architecture of the yeast methyltransferome. **(A)** Correlation-based hierarchical cluster analysis. Each row/column represents a genetic interaction profile for a specific MT. A subset of positive score interactions among the components of COMPASS complex is shown. Strong positive and negative correlations among MTs are indicated by red and blue colors, respectively. **(B)** Scatter plot of the correlation coefficients of *swd1*Δ and *swd3*Δ with their genetic profiles. The most similar profiles for components of COMPASS complex are labeled. **(C)** Correlation-based clusters of similar MT profiles (r > 0.5). Nodes are individual MTs, and edges are correlation coefficient-based interactions between two nodes. Nodes are colored according to a substrate type (red, tRNA; blue, protein; purple, histone, green, small molecule; black, unknown). Edge width and color (red > 0.5, grey < 0.5) represent the magnitude of similarity.

It is well-established that genes encoding proteins that act in the same biological process tend to share the same genetic interactions [11, 12]. This functional relatedness is apparent in the methyltransferome following hierarchical clustering of MTase genetic interaction scores **(Figure S5A)**. For example, we found striking patterns of genetic interactions between MTase members of the COMPASS complex [29], with many exhibiting strong positive interactions. An additional robust predictor of shared function is the correlation between genetic profiles. Correlation of MTase double-mutant fitness profiles genetic profiles revealed that the yeast methyltransferome has a modular structure **(Figure 3A)**. For example, similar to that observed for score-based clustering, correlation-based clustering demonstrated that COMPASS complex members (*SWD1, SWD3, SET1, SDC1,* and *BRE2*) clustered together as their patterns of genetic interactions are much more similar to each other versus other MTases. For example, the correlation of the genetic profile of *swd1*Δ mutant and *swd3*Δ is quite high (*r* =0.83) **(Figure 3B)**.

Although we found extensive evidence for between-cluster genetic interactions, the known (or putative) substrates for each MTase were also reflected in the architecture of the methyltransferome. Specifically, we found enrichment for gene pairs that tend to act on similar substrate types among the MTases with similar genetic profiles (*r >*0.5) (32% of highly similar gene pairs (235) vs. 28% for all gene pairs (3160), *p-*value <0.016, hypergeometric test). Correlation-based clustering revealed that lipid MTases (*OPI3, CHO2*)*,* cofactor MTases (*MET1* and *MHT1; DPH5* and *NNT1*) and protein MTases (*RKM3* and *DOT1*; COMPASS) all clustered together **(Figure 3C)**. These gene pairs were also significantly enriched for positive genetic interactions (score >2.5, *p*-value <1x10^-13^, hypergeometric test). In general, we noted a slight positive correlation between the correlation coefficient and the genetic interaction scores between most MTase gene pairs (*r =* 0.27, *p-*value <1x10^-34^) **(Figure S5B)**. However, there are notable exceptions to this trend. For example, the phospholipid MTases *OPI3* and *CHO2* share similar genetic profiles (*r =*0.64) yet they manifest a strong negative genetic interaction (score < -30). This finding is consistent with negative genetic interactions occurring between genes in the same pathway and with the observation that *OPI3* acts directly downstream of *CHO2* in the yeast phospholipid biosynthetic pathway.

### Relating methyltransferome genetic interactions with physical interactions

**Figure 4:**
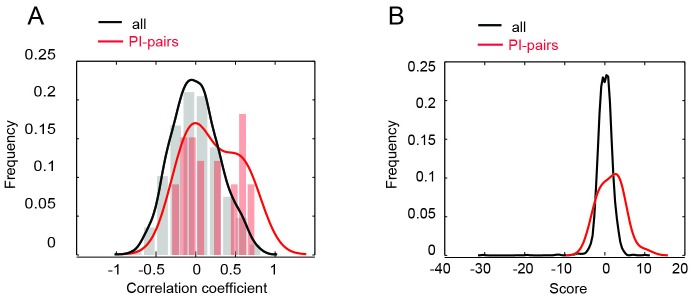
FIGURE 4: Relationship between genetic and physical interactions. **(A)** Distribution of correlation coefficients for all gene pairs (black line) and physically interacting (PI) pairs (red line). **(B) ** Distribution of genetic interaction scores for all gene pairs (black line) and physically interacting (PI) pairs (red line).

In addition to acting in the same biological pathway, genes with similar genetic interaction profiles enriched for strong positive interactions often physically interact [17, 35]. Indeed, in our dataset genes of the COMPASS complex (*BRE2, SDC1, SET1, SWD1, SWD3*), the tRNA MTase complex (*GCD14* and *GCD10*)*,* and two members of the 90S pre-ribosome complex (*NOP2, DIM1*) follow this pattern. Overall, in our screen we found that the MTase pairs encoding physically interacting proteins have similar genetic profiles (r>0.5) more frequently than all interrogated gene pairs **(Figure 4A)**. We also found that these physically interacting gene pairs manifest significant positive and negative scores more often than all tested gene pairs **(Figure 4B)**. In total, 19 out of 54 gene pairs (35%) encoding MTases known to physically interact exhibit significant genetic interactions in our dataset. Furthermore, 7.8% of all MTase gene pairs with significant genetic interaction scores encode for the proteins that associate physically (versus 0.9% for a random set of non-essential genes), suggesting that the methyltransferome is enriched for members that physically interact. It is important to note that despite this enrichment for physical interactors, the majority of genetic interactions (both positive and negative) among MTases do not occur among physically interacting proteins, suggesting that the majority of these genetic interactions represent between-pathway functional relationships versus within-complex interactions. Indeed, consistent with other studies on gene/protein families, we found that gene pairs comprising an essential gene and encoding for physically interacting proteins show a lack of correlation and demonstrate negative genetic interactions relative to the gene pairs composed of only nonessential genes **(Figure 4C)** [21, 30]. For example, in the methyltransferome two of four gene pairs with significant negative genetic interaction had one essential gene (*SWD2* or *NOP2*) versus 0 of 14 gene pairs with positive genetic interactions. One interpretation for these observations is that complexes containing essential components are more vulnerable to additional genetic perturbation. The fact that we can detect such patterns that were previously detected in larger, non-targeted genome-wide studies validates the predictive nature of our dataset.

### Plasticity of the methyltransferome in environmental stress

**Figure 5:**
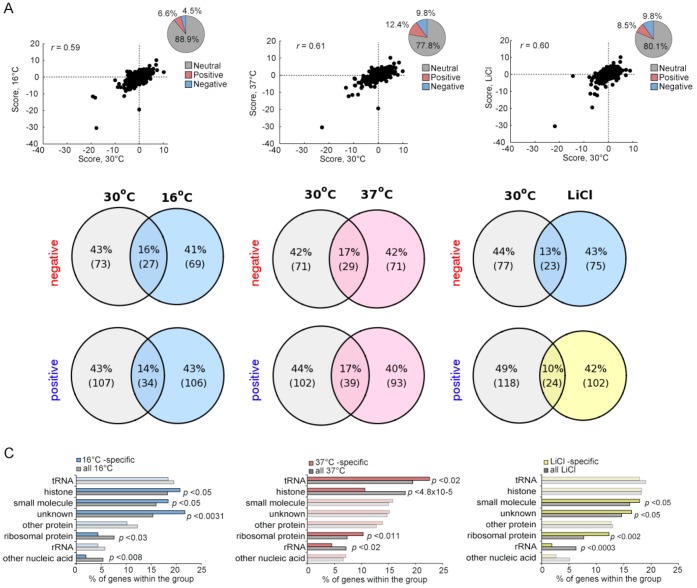
FIGURE 5: Plasticity of the methyltransferome in response to stress. **(A)** Genetic interactions for MT gene pairs under stress conditions (16°C, 37°C or LiCl) plotted against the those at 30°C. Insets: percentage of each interaction type. **(B)** Overlap of significant genetic interactions (|score| >2.5) between 30°C and the indicated stress conditions **(C)** Enrichment for genetic interactions between gene pairs by substrate type for stress conditions.

Cells exposed to stress maintain their homeostasis, in part, by altering their transcription, translation and signaling pathways [16]. Given that certain genetic interactions are condition-dependent, we and others have shown that genetic interaction networks can be stress specific and that the patterns of strain sensitivity in the face of such stressors can reveal details of each response pathway [25, 31, 32]. To evaluate the environmental-dependence of the yeast methyltransferome network we quantified the fitness of all digenic MTase mutants in three environmental stress conditions: 1) 16°C, 2) 37°C and 3) 0.25 mM LiCl. While the proportion of the significant positive and negative genetic interactions (|score| >2.5) observed in the methyltransferome stress networks were similar to the ~25% observed in the 30°C reference condition **(Figure 5A)**, we found stress-specific genetic interactions **(Figure S6)**. For example, 106 significant positive interactions identified at 16°C (~75%) were not detected in the reference sample **(Figure 5B)**. The stress-specific gene interactions were represented by particular substrate-type MTases **(Figure 5C)**. For example, the 16°C- and LiCl-specific genetic interaction datasets were enriched for unknown MTases (*p*-value <0.003 and *p*-value <0.05, respectively) and for small molecule MTases (*p*-value <0.05). In contrast, the 37°C-specific interacting gene pairs were enriched for tRNA and ribosomal protein MTases (*p*-value <0.02 and *p*-value <0.011), with histone MTases being substantially underrepresented (*p*-value <4.8x10^-5^). Furthermore, rRNA MTases were underrepresented in both LiCl- and 37°C-specific datasets.

### A core MTase network

**Figure 6:**
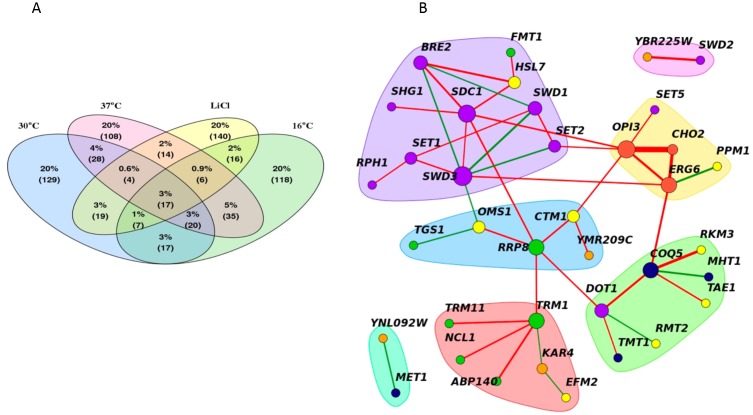
FIGURE 6: A core methyltransferase network. **(A)** Venn diagram of the shared significant interactions between all four conditions (30°C, 16°C, 37°C or LiCl). **(B)** The core methyltransferase genetic network shared by at least three of the four conditions tested. Red and green edges denote negative and positive genetic interactions, respectively. Line width represents average genetic interaction strength. Node color represents biological substrate; purple (histone), green (RNA), yellow (small molecule), red (lipid), orange (unknown) and navy (small molecule). Distinct groups were identified by clustering edge betweenness.

Despite the observed changes in the genetic interactions between stress conditions and the reference condition, certain genetic interactions were consistent across all conditions. On average, ~25% of significant negative genetic interactions (either positive or negative) were shared between any single stress network and the reference, with the smallest overlap observed for LiCl positive interactions (19%) **(Figure 6A)**. We used these data to define a core, “conserved” genetic network of 45 MTase gene pairs (significant in at least three of the four conditions (|score|>2.5). This set included small molecule, lipid, RNA, protein, histone and uncharacterized MTases. The core MTase network was significantly enriched for lipid and histone MTases (*p*-value <0.002, hypergeometric test) compared to all significant gene pairs **(Figure 6B)**.

This core MTase network highlights genetic links between diverse MTase coordinated biological processes and basal functional architecture of the methyltransferome. In this network, nodes are represented by genes and edges connect gene pairs with genetic interactions. Highly interconnected nodes form distinct clusters or hubs- several of which were enriched for biological processes. For example, in our network, hubs included specific pairwise interactions between: 1) lipid MTases *CHO2, ERG6, OPI3* 2) tRNA MTases *TRM1, TRM11, NCL1, ABP140* 3) small molecule MTases *MHT1, COQ5, TMT1* and 4) histone MTases *BRE2, SDC1, SET1, SET2, SHG1, SWD1, SWD3, RPH1*. Edges in the network with high “betweenness” (a measure of how important a node is in a network) serve as bridges that connect these hubs and include *OPI3, COQ5, TRM1, SET2 and SWD3.* These edges suggest links between the biological processes represented by the clusters suggesting that they may act to buffer each other.

One notable feature of the core MTase network is that lipid-histone and lipid-small molecule MTases are biologically linked and that alterations in one can impact the other. This feature of our dataset is consistent with published observations that genes involved in sulfur and phospholipid metabolism are coordinately regulated. By way of illustration, the MTases Cho2 and Opi3 comprise key elements of phospholipid biosynthetic pathway. These two MTases are also major consumers of cellular SAM pools and it is known that sufficient flux through this pathway is required to generate the required levels of SAH for proper regulation of sulfur amino acid biosynthetic pathways [33, 34]. The genetic interactions we observe between Cho2 and Opi3 reflect the interplay between these pathways. In addition, the transcriptional regulators of sulfur (Met4) and phospholipid (Opi1) metabolic pathways act in concert to maintain cellular levels of SAM [33].

The genetic interactions between the histone and lipid clusters in our network in agreement with several high throughput studies [12, 24, 35]. Interestingly, a recent study demonstrated *cho2*Δ deletion strains accumulate SAM, and in conditions requiring methionine, lead to the hypermethylation of histones, suggesting that histone MTases may act to reduce SAM levels. Our results suggest a similar crosstalk and, taken together, these studies underscore the importance of continued work on the regulation of intracellular SAM:SAH ratios and the importance of coordination of sulfur metabolism and SAM MTase pathways.

### The COMPASS complex remodels in stress conditions

**Figure 7:**
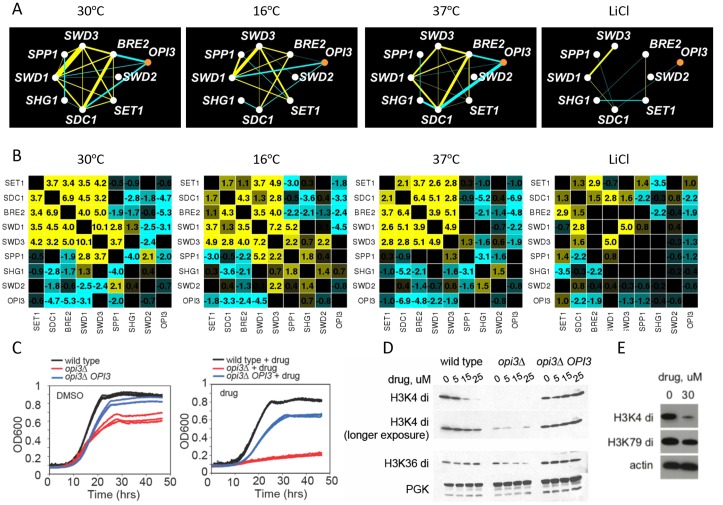
FIGURE 7: Exploring COMPASS and phospholipid MT connection. **(A)** Genetic interactions among COMPASS MTs and *OPI3.* Yellow; positive genetic interaction, cyan; negative genetic interaction. **(B)** Genetic interactions among COMPASS MTs and other genes in the four stress conditions. Yellow; positive genetic interaction, cyan; negative genetic interaction. **(C)** Overexpression of *OPI3* rescues the drug-induced fitness defect *opi3*Δ in the presence and absence of the *opi3*Δ chemical probe. Growth of wild-type, *opi3*Δ and *opi3*Δ overexpressing *OPI3,* without drug (left panel) and with drug (right panel). Growth is measured by as O.D.600 (y-axis) as a function of time (hrs)(x-axis). **(D)** *OPI3* is important for histone methylation. Levels of di-methylated H3K4 and H3K36 in the indicated strains and drug conditions. **(E)** Levels of di-methylated H3K4 and H3K79in in A549 cell in response to 30μM *opi3*Δ chemical probe.

Although the biological modules of functionally related genes are typically conserved among distantly related species and across conditions, it has also been shown that the genetic wiring is reprogrammed in response to stress [31, 36]. In our screen, the core COMPASS complex members (*SET1, SDC1, SWD1, SWD2,* and *SWD3*) [37] exhibited strong positive genetic interactions and highly correlated genetic profiles in the reference condition (30°C) **(Figure 3A)** and to a lesser extent in the 16°C and 37°C stress conditions. In LiCl however, only a subset of subunits of the COMPASS were present, and with the exception of *SWD1* and *SWD3,* their genetic interactions were not conserved **(Figure 7A).** This observation suggests that COMPASS MTases rearrange their genetic interactions in response to this stress. In addition, in the reference condition (30°C) COMPASS components *BRE2, SDC1 and SWD1*, exhibited negative genetic interactions with the phospholipid MTase *OPI3* **(Figure 7A)**, while at 16°C they did not, suggesting that some MTase genetic interactions in COMPASS are stress-responsive **(Figure 7A and Figure 7B)**. Additionally, their genetic profiles were not similar (i.e. they showed low correlation), suggesting these MTases function independently. In particular, in LiCl stress conditions there was a substantial loss of positive interactions within the complex in the stress conditions accompanied by loss of the COMPASS cluster in the heatmap **(Figure S7)**. At 37°C and in LiCl the genetic interaction profiles of the majority of COMPASS components clustered with *SET2* (histone lysine K36 MTase) and the phospholipid MTase *CHO2*, suggesting a condition-dependent functional link between these enzymes under these conditions. These observations indicate that COMPASS is a dynamic complex that remodels in response to environmental stress.

### Exploring the connection between COMPASS and phospholipid MTase

To further explore the link between phospholipid and histone MTases observed in our dataset, we used a *opi3*Δ specific chemical probe uncovered in our large-scale genome-wide screening effort [38] (SGTC 2241) **(Figure S8)**. This compound ((5E)-5-[(4-hydroxyphenyl)methylidene]-3-prop-2-ynyl-1,3-thiazolidine-2,4-dione; PubChem CID:2285411) induced drug-sensitivity in the *opi3*Δ deletion strain, suggesting that Opi3 is required to resist the effects of this compound. Specifically, i) the *opi3*Δ deletion strain exhibited a drug-induced fitness defect and ii) dose-dependent overexpression of Opi3 increased resistance to this compound **(Figure 7C)** encouraging our efforts to use compound 2241 as a chemical probe of the *OPI3* pathway function.

We found that treatment of wildtype cells as well as cells with altered Opi3 gene dose with compound 2241 resulted in a dose-dependent reduction of histone methylation (H3K4di and H3K36) **(Figure 7D)** as assessed by immunoblot with methylation-specific histone H3 antibodies. This effect was *OPI3* specific because overexpression of Opi3 protein in both mutant and wildtype cells rescued the phenotype suggesting that chemical inhibition of Opi3 affects the methylation status of histones **(Figure 7D and Figure S9)**. Therefore, both chemical and genetic perturbation of reduction of Opi3 levels reduces histone methylation.

To ask if the effects of compound 2241 were conserved in mammalian cells, human lung carcinoma (A549) cells were exposed to this compound and assayed for growth inhibition and histone H3 methylation status. We observed an analogous dose dependent inhibition of histone methylation (H3K4di) **(Figure 7E and Figure S9)**. The observation that perturbation of a phospholipid MTase (Opi3) leads to changes in the methylation state of histones is intriguing, in light of the fact that methylation of phosphatidylethanolamine (PE) serves as a major consumer of SAM in the cell.

## DISCUSSION

The analysis of genetic interactions in the yeast methyltransferome network demonstrates our dataset is of high quality and is comparable to other established high-throughput datasets [12, 24, 35]. Several properties of genome-scale genetic networks were recognizable in the methyltransferome, including the higher frequency of interactions observed for slow-growing MTase deletion strains and the bias towards positive interactions between genes encoding physically interacting proteins.

The enrichment for genetic interactions among MTases is consistent with other functionally focused genetic interaction studies [17, 24, 25] and the inclusion of additional environmental stressors highlights the plasticity of the methyltransferome network. Though the frequency of interactions remained relatively constant in response to stress, a substantial number were stress-specific. For example, different stresses exhibited slight enrichments for interactions by substrate. Notably, interactions that were conserved in sign between the reference and stress conditions were significantly dampened, suggesting that the magnitude and not just the differences between condition-dependent interactions should be considered. Our observations also suggest that additional functional information will be gleaned by using more specific stresses in order to realize the full extent of functional redundancy among MTases. For example, in a DNA repair focused SGA study, the number of interactions doubled in the presence of DNA-damaging agents [17, 24, 25]. Choosing such precise perturbations a priori for genesets linked only by enzymatic activity is not as straightforward as those linked by function. However, the methyltransferome revealed vulnerabilities that can be exploited in future studies. For example, because lipid MTases are highly connected hubs in our network, drugs or stressors targeting their metabolic pathways or function are likely to be functionally informative.

The plasticity of the yeast methyltransferome is exemplified by interactions with the COMPASS complex. COMPASS (Complex Proteins Associated with Set1) is an evolutionary conserved histone 3 lysine 4 (H3K4) methyltransferase complex comprised of seven polypeptides and a highly conserved ~140 amino acid SET domain [29]. At 30°C, core members of COMPASS, including the catalytic subunit *SET1* and the regulatory and structural components *BRE2, SDC1, SWD1* and *SWD3*, shared strong positive interactions and tightly clustered genetic profiles in agreement with the subunits known to be required for histone H3K4 methylation [39]. In contrast, the remaining subunits including *SWD2*, an essential gene, *SPP1*, and *SHG1*, exhibited genetic interactions distinct from each other and the core complex, consistent with their roles in diverse processes [37, 40]. In response to stress, genetic interactions between the core COMPASS subunits were significantly dampened at 16°C and 37°C, and the correlation between profiles completely lost in LiCl. It is possible that the complex disassociates or is simply not required in LiCl. It would be interesting to test whether the functional dissolution is reflected at the level of these components' physical interactions.

Genetic interactions between the COMPASS complex members (*BRE2, SDC1, SWD1* and *SWD3*) exhibited strong negative interactions with the protein MTase Hsl7 at 30°C. These interactions were partly lost at 16°C and 37°C, and completely lost in LiCl, suggesting network rewiring is required to resist stress. Hsl7 is an arginine protein MTase involved in cell cycle regulation. Although recombinant Hsl7 demonstrates enzymatic activity towards histones H2A and H4 *in vitro* [41], *in vivo* histone methylation has not been not detected, suggesting that Hsl7 may be active against histone proteins only under certain conditions [42]. Consistent with these observations, HSL7 is synthetically lethal with SET1 and other chromatin-remodeling enzymes, and can act as a transcriptional regulator [43]. The Hsl7 human homolog, Prmt5, is linked to cancer through its activity as a transcriptional repressor. These results suggest that Hsl7 may buffer COMPASS activity by methylating additional, as yet to be identified, targets.

Our genomic analysis of the yeast methyltransferome, combined with chemical-genetic analysis of the relationship between histone and phospholipid MTases, allowed us to characterize a new role for Opi3 in mediating histone methylation. We found histone methylation of both H3K4 and H3K36 were dramatically reduced in *opi3*Δ and wildtype cells, a phenotype further exacerbated by treatment with the *opi3*Δ-specific chemical probe. Overexpression of Opi3 restored histone methylation to wildtype levels and all cases, demonstrating its specificity. These results suggest that Opi3 and histone methylation may be coordinately regulated. In further support for this crossstalk, we found significant correlation between chemical-genetic fitness profiles measured across > 3000 small molecules (p-value < 1e-03) between *OPI3* and COMPASS complex members *BRE2, SDC1, SWD1* and *SWD3*) [44].

A recent report supports a metabolic link between phospholipid metabolism and histone methylation [45]. Under conditions of methionine starvation, deletion of the phospholipid *CHO2* MTase accumulated high levels of SAM. The disruption of the SAM/SAH ratio promoted histone methylation, leading the authors to conclude that histone MTases act as methyl sinks to meet the cellular demand for SAH required for methionine biosynthesis. Although the hypermethylation in the *cho2*Δ seemingly contradicts the hypomethylation we observed in *opi3*Δ, a number of factors may account for tipping the balance of the cellular response in the opposite direction. These include the experimental differences in genomic, environment and epigenetic context and cellular state. Whatever the mechanism, we suggest that the differences in particular methylation marks are less important than the crosstalk between lipid and histone methylation. Deciphering the histone code is notoriously difficult as methylation marks are complex and dynamic; hypomethylation at one site can promote hypermethylation at another to compensate [45]. Previous work has established metabolic coordination between sulfur and phospholipid metabolism [33, 34]. Our work extends these findings by adding histone methylation to the interplay between lipid and SAM biology. Future work on the mechanism by which Opi3 (and its human homolog PEMT) act to modify histones and impact SAM will likely illuminate the mechanism of this interplay in both normal and pathological states, and how the histone code context affects these pathways.

Our systematic analysis of the yeast methyltransferome provides a valuable resource for increasing our understanding of methyltransferase function by: 1) serving as a benchmark in the form of the first global methyltransferome genetic interaction compendium 2) identifying condition-specific interactions 3) revealing network vulnerabilities that may have potential as therapeutic targets and 4) identifying conserved features that can be leveraged in focused human genetic interactions studies.

## MATERIALS AND METHODS

### Strains and growth conditions

Single-deletion query strains (*MTaseΔ::Nat*^*R*^ ) were kindly provided by the Boone lab [12]. Essential alleles used as queries in this study were previously made in our lab using a DAmP (decreased abundance by mRNA perturbation) strategy [46]. To construct all pairwise double-deletion MTases query strains were mated with the yeast deletion collection (*MTaseΔ::Kan*^*R*^) [47] using the Synthetic Genetic Array (SGA) protocol as described [14, 20]. The generated double mutants were maintained in YPD containing geneticin (G418) and nourseothricin (NAT) and stored at -80°C. For growth tests, cells were grown in YPD containing G418 and NAT or SD to mid-exponential phase and diluted to OD_600_ 0.2. Liquid growth assays were performed using TECAN GENios microplate reader 30°C [48].

### Construction of double-deletion mutants

In brief, we designed an ordered (by systematic name) array containing 82 MTase deletion mutants (*MATa* haploids), where each MT gene is replaced with the kanamycin resistance marker (Kan^R^). The array was consolidated into a 1536 colony format by pinning three sets of MTase arrays onto one plate where each mutant is represented four times. To minimize the well-known technical variation of colony sizes due to plate location effects, two outermost rows and columns, as well as four columns between the array sets were filled with control *his3*Δ strain. Next, using SGA technology [11, 12, 49] we crossed the arrayed MT mutants with deletion query mutants (*MAT*α haploids) where the MTase gene is replaced with the nourseothricin resistance marker gene (Nat^R^). A series of robotic pinnings (BM10, S&P Robotics, Inc.) of the arrays on selective media was used to induce meiosis and select for haploid mutants carrying both deletions. In total, the set of 33 plates contained 7614 double-deletion mutants, where each mutant was represented by at least four replicates. The reciprocal mutants (over 80% in a set) were represented by 8 replicates (KanR-NatR and NatR-KanR pairs). Haploid double-mutant colonies were photographed after a defined interval (2-3 days), and colony sizes were quantified using ColonyImager software [49].

### Histone methylation assays

To assay histone methylation status, cells were treated with the *opi3*Δ chemical probe for 2 hours (hrs) in SC media, lysed and analyzed by western blotting using H3K4 and an H3K36 di-methyl specific antibodies. Phospho-glycerol kinase (PGK) was used as a loading control. In the mammalian assays, following treatment with 30 µM of *opi3*Δ chemical probe for 2hrs, cell lysates from A549 cells were separated by SDS-PAGE, immunoblotted and probed with H3K4 and H3K79 di-methyl specific antibodies. Actin was used as a loading control.

### Data analysis and genetic interaction score

The procedure for scoring genetic interactions was adopted from [22]. Colony sizes were first normalized to correct for systematic artifacts. Briefly, normalization steps included scaling each colony size to 1) the median of all double mutants carrying this NAT-marked mutation 2) the median of all double mutants in a particular set and 3) the median of all double mutants carrying this KAN-marked mutation within the same position on the plate. This normalization procedure corrects for both growth differences between plates as well as growth defects associated with a given query strain.

To calculate a robust genetic interaction score, several systematic technical and quality control steps must be corrected for. The final interaction accounts for the difference in the medians of the normalized sizes of the double mutants and their expected sizes, divided by the sum of their standard deviations. The score was obtained using the following equation:

(1) $$Score=\frac{\mu_{double}-\mu_{control}}{\sqrt{\frac{S_{\mathrm{var}}}{\eta_{double}}+\frac{S_{\mathrm{var}}}{\eta_{control}}}}$$

where: *μ_double_* is the mean of normalized colony sizes for the double mutant of interest; *μ_control_* is the median of normalized colony sizes for all double mutant of containing the Kan-marked mutant of interest (array); *η_double_* and *η_control_* is 4 (number of experimental replicates); *S*_*control*_ is the median of the variances in normalized colony sizes observed for all double mutants containing the Kan-marked mutant of interest (array); *S*_*var*_ is the variance of the normalized colony sizes for the double mutant of interest defined as:

(2) $$S_{\mathrm{var}}=\frac{\sigma_{double}^{2}(\eta_{double}-1)+\sigma_{control}^{2}(\eta_{control}-1)}{\eta_{double}+\eta_{control}-2}$$

where *σ^2^_double_* is the maximum of the variance of normalized colony sizes for the double mutant of interest; *σ^2^_countrol_* is the median control of the variances in normalized colony sizes observed for all double mutants containing the KAN-marked mutant of interest. Because the double mutant strains in which the markers (NatMX and KanMX) are present in the same chromosomal locations the double mutant is lethal (i.e. the strains on the diagonal in the matrix) due to inability to grow in the presence of both selectable markers (G418 and NAT), we used this phenotype to check for the accuracy of the strains. Additionally, to decrease the rate of false positive scores for the genes located within 50kb of each other, these gene pairs were also filtered out **(Figure S1C)** due to a possible linkage of these genes that may result in failure to segregate during meiosis. We also found that the genetic profiles of the reciprocal strains (NatR-KanR vs KanR-NatR) tend to correlate strongly to each other **(Figure S1D)**. Based on these measures we were able to identify incorrect strains. For the final calculations of the scores, we removed reciprocal gene pairs with opposite signs and averaged the scores with the same sign. The scores for the gene pairs present only in “one direction” in the screen were halved, so they would be comparable to the averaged scores for the reciprocal strains.

### Data analysis

Data manipulation and statistical analysis was performed with MATLAB and R [50]. Hierarchical clustering analysis was performed using Cluster 3.0 using average linkage as the clustering method. Euclidean and Pearson correlation were used as the distance metric for genetic interaction and correlation-based networks, respectively. Heatmap figures were created using Java Treeview [51] or R [50].

## SUPPLEMENTAL MATERIAL

Click here for supplemental data file.

All supplemental data for this article are available online at http://www.microbialcell.com/researcharticles/2019a-giaever-microbial-cell/.

## Abbreviations:

MTase: – methyltransferase,
SAH: – S-adenosylhomocysteine,
SAM: – S-adenoxylmethionine,
SGA: – Synthetic Genetic Array.
