# Supplementary material for: Network dynamics of the yeast methyltransferome
Source: Microb Cell. 2019 Jul 9;6(8):356–69. doi: 10.15698/mic2019.08.687 (PMC6685046; doi:10.15698/mic2019.08.687)
Supplement: Supplementary file 1 [file mic-06-356-s01.pdf]

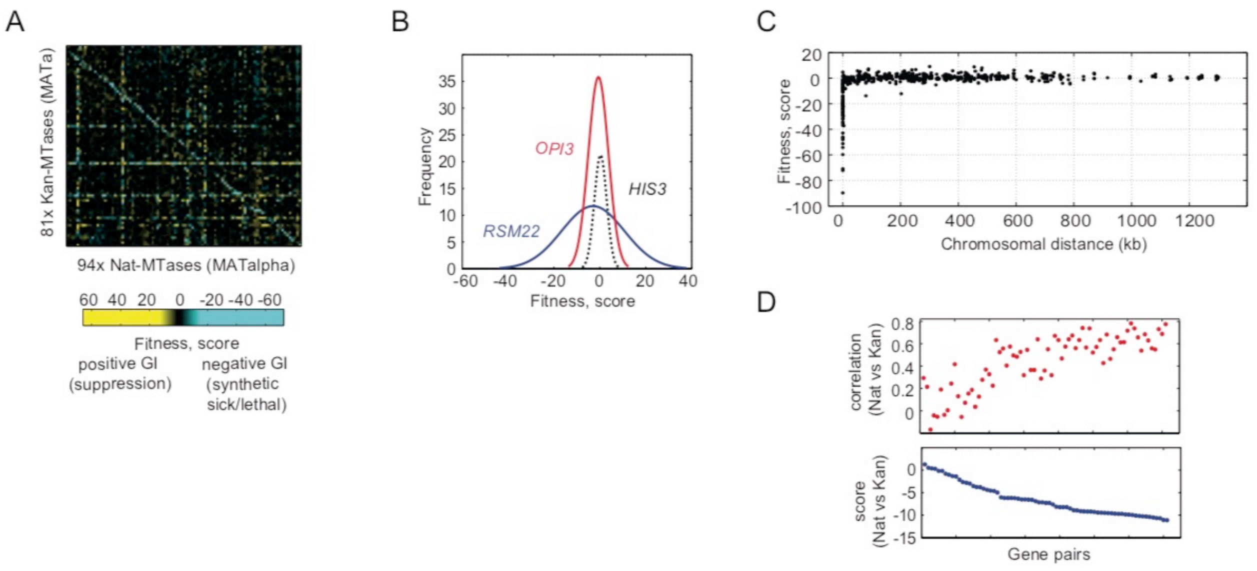

Figure S1.

(A) Correlation-based hierarchical cluster analysis. Each row/column represents a genetic interaction profile pattern for a specific MTase. A subset of positive score interactions among the components of COMPASS complex is shown. Strong positive and negative correlations among MTases are indicated by yellow and cyan, respectively.

(B) Slow-growing query strains (*OPI3Δ*, *RSM22Δ*) have a greater number of significant genetic interactions (y-axis, frequency) and/or more extreme positive or negative fitness scores compared to non-slow growing (*HIS3Δ* control).

(C) Raw, unaveraged genetic interaction scores for a given gene pair as a function of the chromosomal distance between these two genes in kilobases (kb). Strains with linked genes (within 50 kb) were removed from the analysis.

(D) Plots demonstrating the influence of the correlation between reciprocal pairs (Kan^R-Nat^R vs Nat^R-Kan^R) on genetic interaction score.

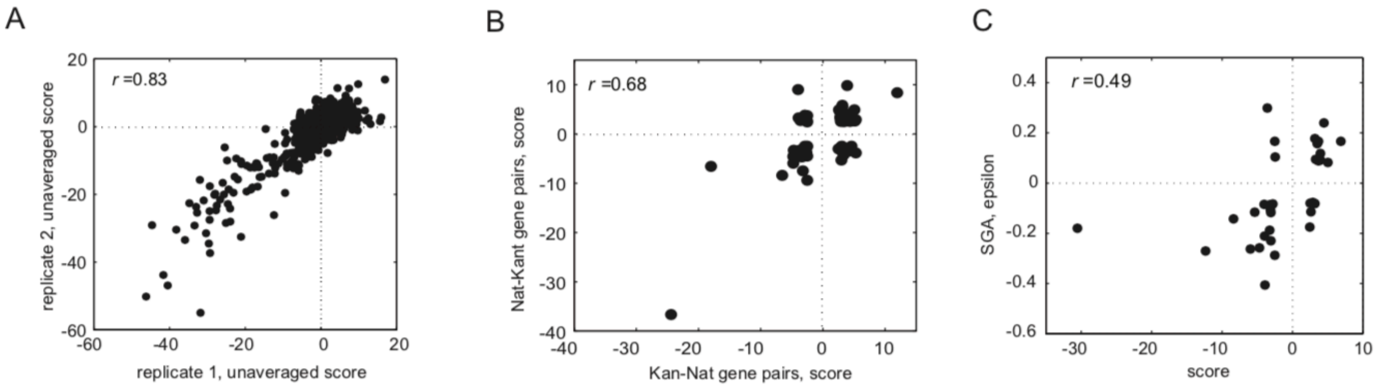

Figure S2. Reproducibility of genetic interactions

(A) Correlation of interaction scores between two independent screens.

(B) Correlation of high-confidence genetic scores (|score| >2.5) between independently constructed reciprocal strains (Nat^R-Kan^R and Kan^R-Nat^R) after removal of those with opposite signs.

(C) Estimate of false discovery rate. Overlap of methyltransferase genetic interactions in our study with a large-scale genome-wide SGA study ($|\varepsilon| > 0.08$, p-value < 0.05) (Costanzo et al, 2010)($r = 0.49$, p-value < 0.003).

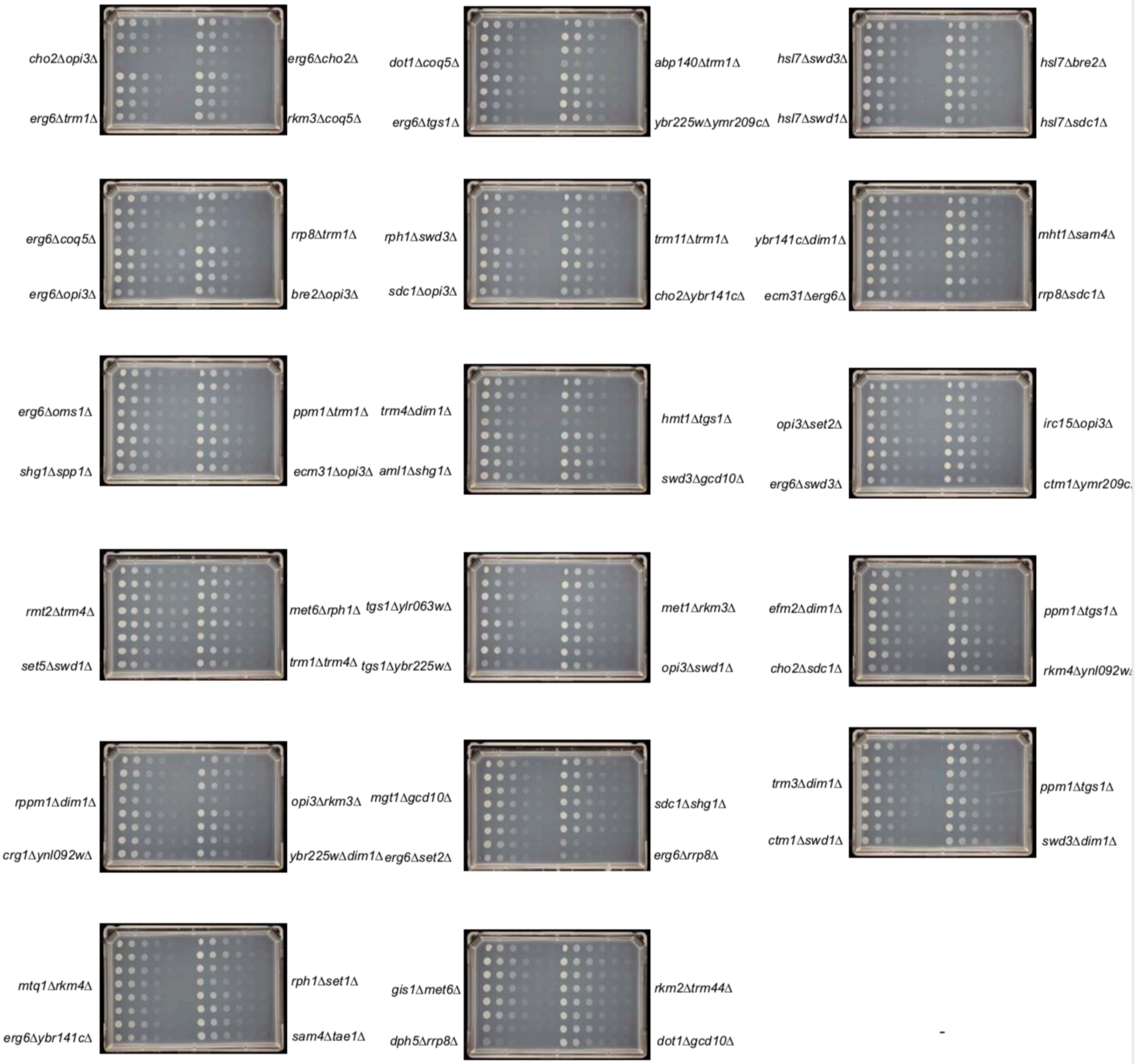

Figure S3. Evaluation of a subset of significant negative genetic interaction scores using serial dilution spot assays. The growth of each strain is indicated in groups of four where the first row is wildtype, the fourth row is the double MTase mutant and the second and third row the first and second single mutant strain of the labeled pair, respectively. Saturated cultures were serially diluted (in 5-fold steps) into a 96-well plate and ~5μl from each well was transferred to agar plates using a pin tool. Cells were grown for 48hrs and then photographed. We estimate our false positive rate as ~40% for negative genetic interactions by visual inspection.

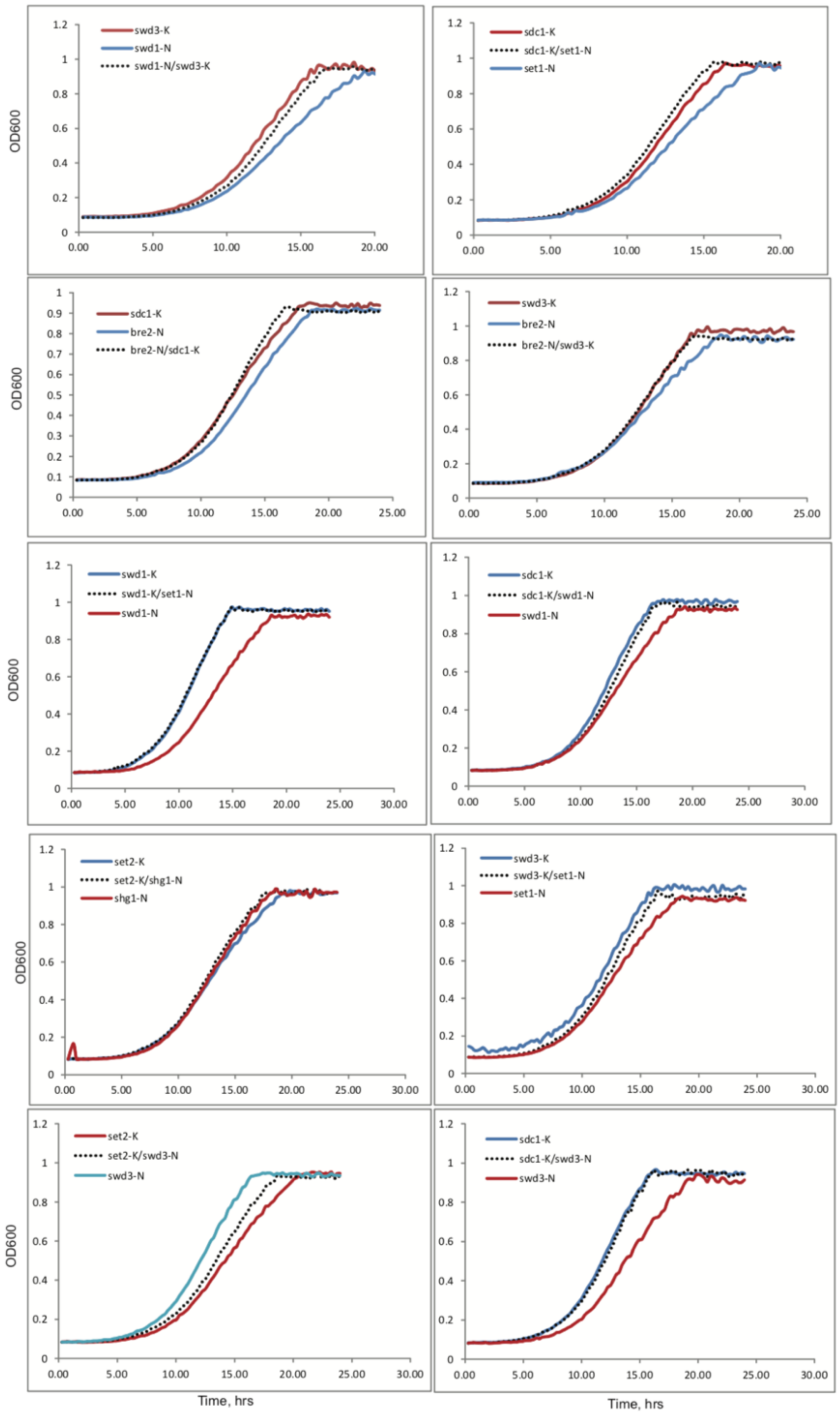

Figure S4. Confirmation of a subset of significant positive interaction scores using high resolution liquid growth assays. Single mutant growth rates were compared to the growth of their double mutant counterparts using high resolution liquid growth assays in which 100μl cultures are assayed for O.D.$_{600}$ at 30° C every 15minutes. Genotypes including the drug resistant markers kanamycin (-K) for query strains and neomycin (-N) for the array strains are indicated. Optical density (O.D.$_{600}$) (y-axis) plotted as a function of time (hrs) (x-axis). Confirmed positive genetic interactions from left to right include the double mutants: 1) *swd1Δ swd3Δ* 2) *sdc1Δ set1Δ* 3) *bre2Δ sdc1Δ* 4) *bre2Δ swd3Δ*, 5) *set1Δ swd1Δ*, 6) *sdc1Δ swd1Δ*, 7) *set2*^{damp} *shg1Δ,* 8) *set1Δ swd3Δ* 9) *set2*^{damp} *swd3Δ* and 10) *sdc1Δswd3Δ.* We estimate our false positive rate as ~50% for positive genetic interactions by visual inspection.

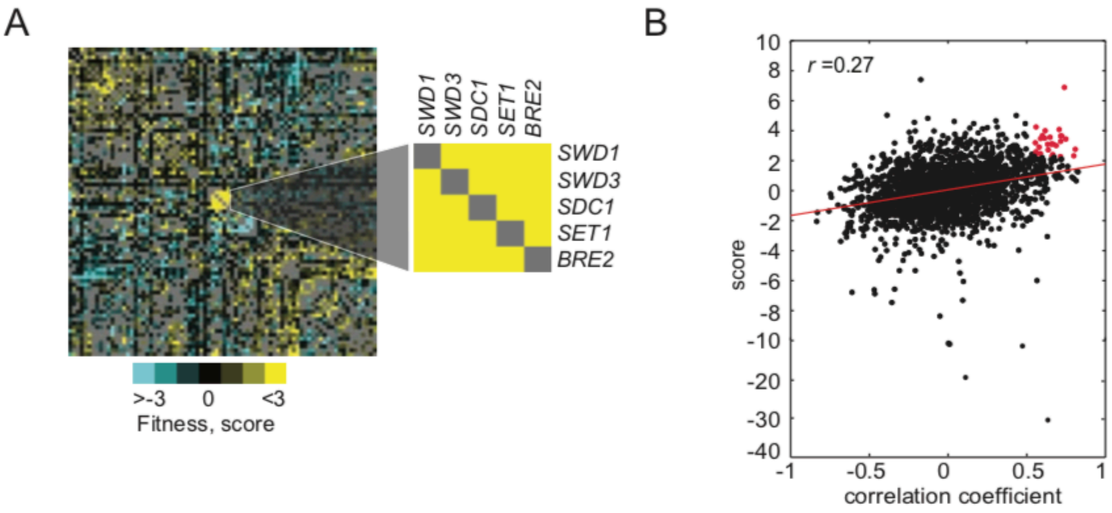

Figure S5.

(A) Hierarchical clustering of genetic interaction scores.

(B) Correlation between genetic interactions score and correlation coefficient. Genetic interaction scores for MT gene pairs (y-axis) as a function of the Pearson correlation coefficient (x-axis) ($r = 0.27$, p-value $<1e^{-34}$).

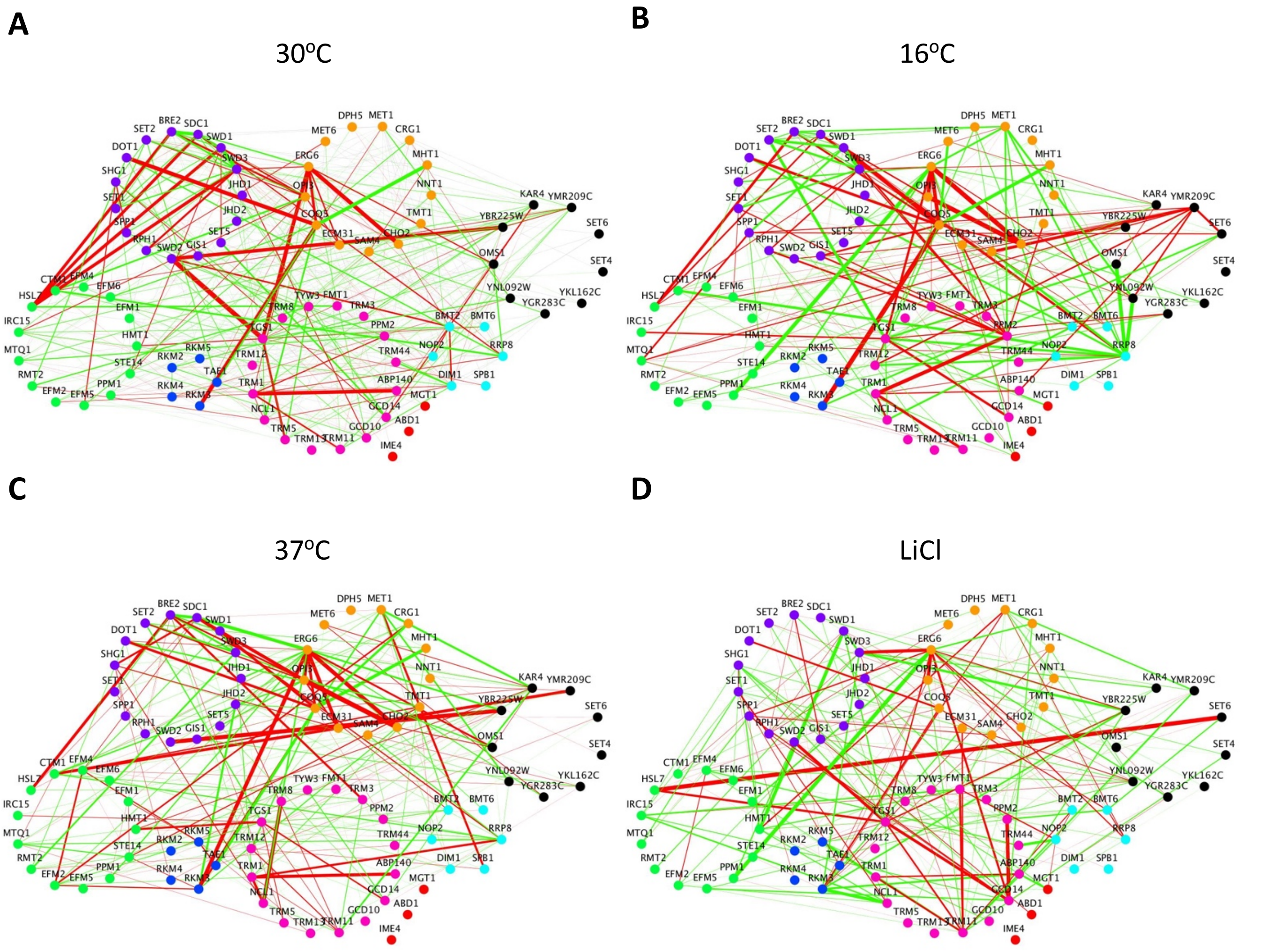

Figure S6. Remodeling of the MTase network: Genetic interactions change in response to stress

Each MTase gene is represented as a node in the network and significant genetic interactions are represented by edges. Nodes are colored according to substrate type. Edge width represents the strength of the genetic interaction score and edge color the interaction type (red negative, green positive).

(A) 30°C

(B) 16°C

(C) 37°C

(D) 0.25mM LiCl

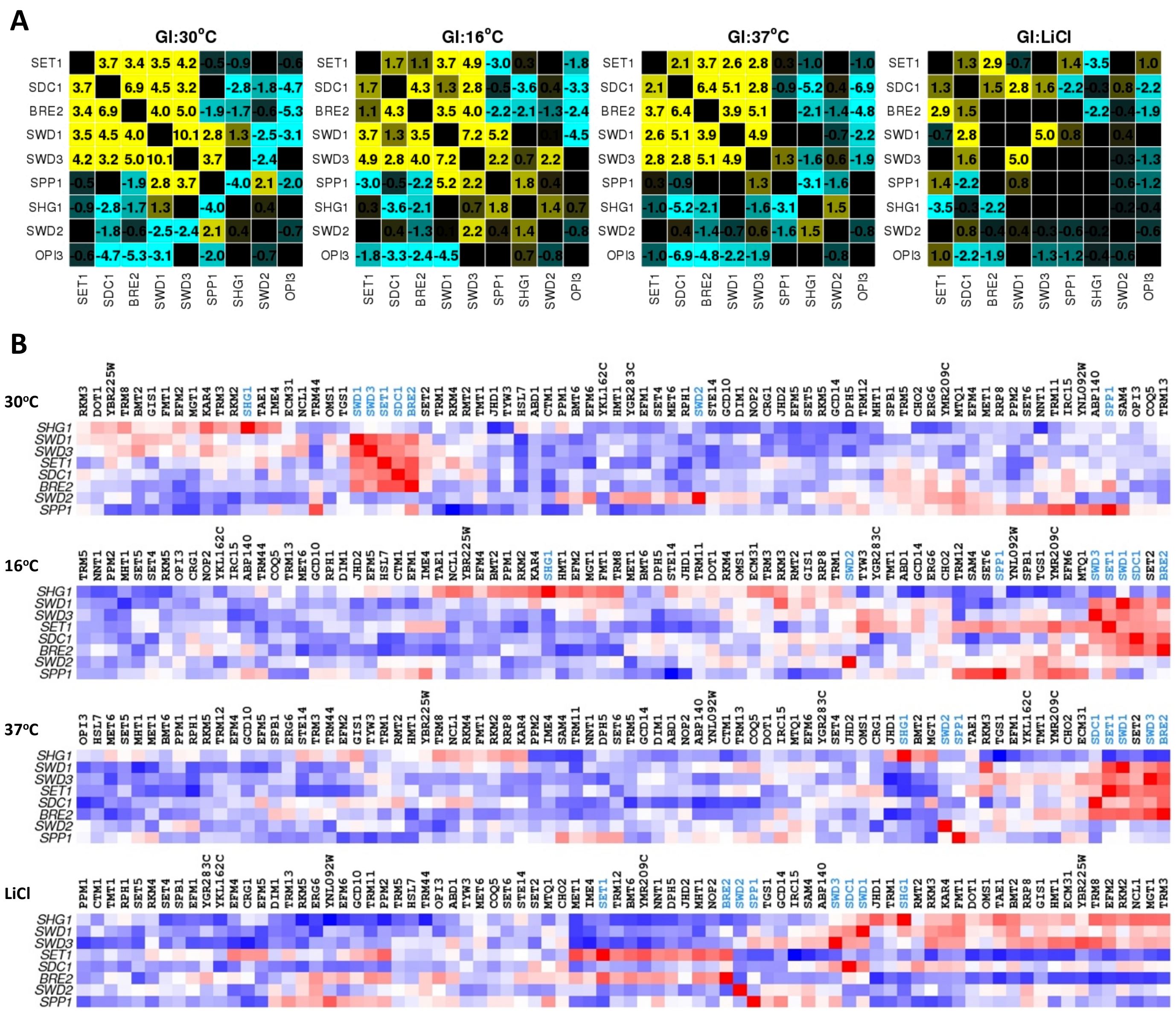

Figure S7. Characterizing the COMPASS complex in response to stress.

(A) Genetic interactions between members of the compass complex in the four stress conditions.

(B) Correlation of genetic profiles of the COMPASS genes at 30°C, 16°C, 37°C, and 0.25mM LiCl.

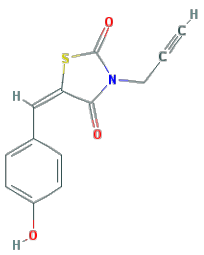

Figure S8. Structure of SGTC_2241 *OPI3* small molecule sensitizer
PCID 2285411

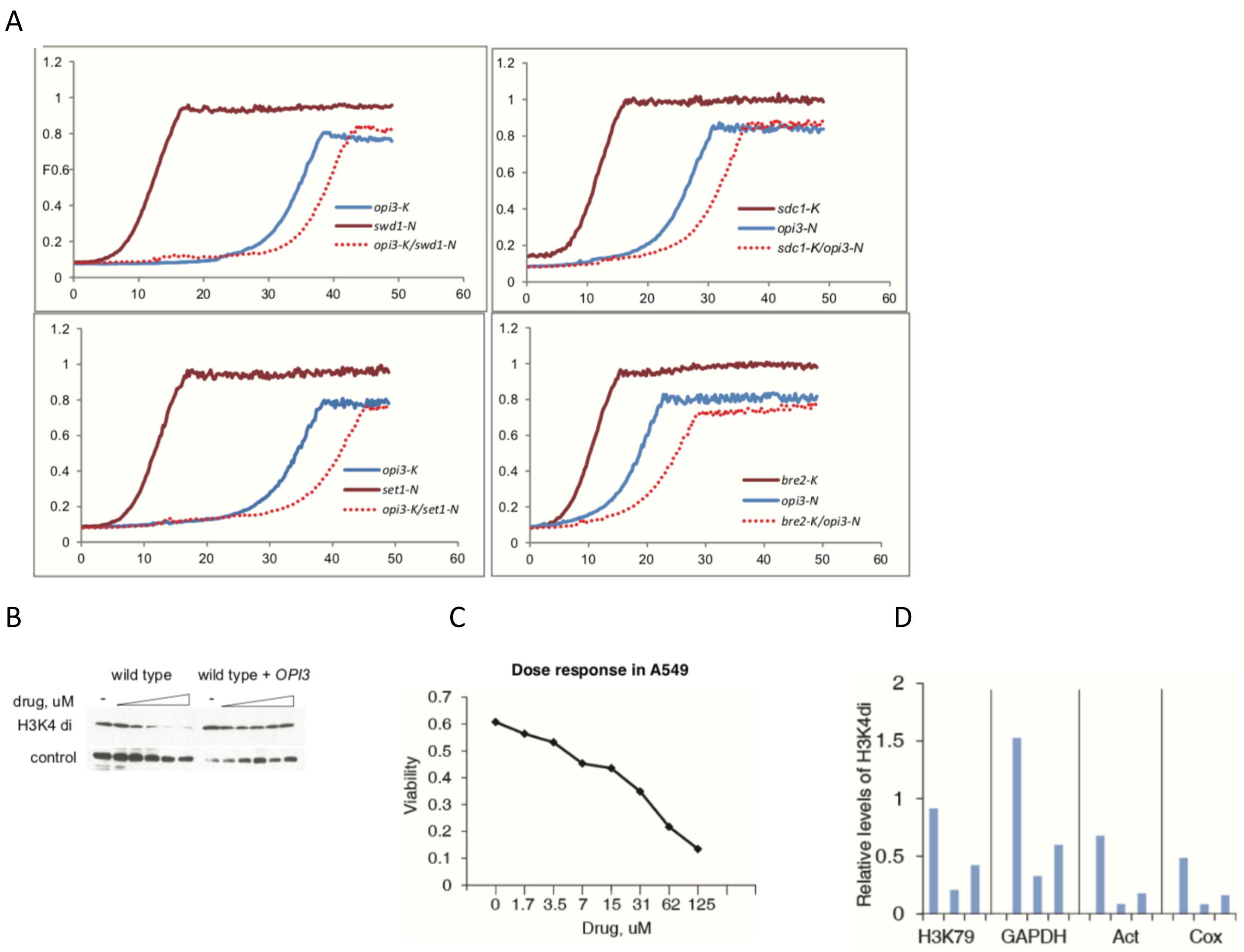

Figure S9. Interactions between members of the COMPASS complex.

- **(A)** Confirmation of negative genetic interactions between *opi3Δ* and several deletions of COMPASS complex members (*swd1Δ*, *sdc1Δ*, *set1Δ* and *bre2Δ*). Single mutant growth rates were compared to the growth of the double mutant counterpart. Genotypes include the drug resistant markers kanamycin (-K) for query strains and neomycin (-N) for the array strains. Optical density (O.D.$_{600}$) (y-axis) plotted as a function of time (hrs) (x-axis).
- **(B)** *OPI3* gene dose is important for histone methylation (H3K4). Wild-type, and wild-type overexpressing *OPI3* were collected after the treatment with the compound for 2 hours in SC media. The cell lysates were analyzed by western blotting with anti-H3K4 di methyl antibody. Anti PGK was used for an internal loading control.
- **(C)** Dose response of A549 cells in response to the Opi3 sensitizer drug PCID 2285411.
- **(D)** Quantitation of immunoblot data for relative levels of histone H3 K4 dimethylation. The each set of three bars correspond to Wild-type, opi3 deletion strain and opi3 deletion overexpressing OPI3.

Filename: april1_sub_suppfigs.docx
Directory: /Users/gurinina/Dropbox/Documents/2018_elena/2019_mar10_elena/mar30_final
Template: Normal.dotm
Title:
Subject:
Author: ggiaever
Keywords:
Comments:
Creation Date: 4/1/19 9:37:00 AM
Change Number: 2
Last Saved On: 4/1/19 9:37:00 AM
Last Saved By: ggiaever
Total Editing Time: 1 Minute
Last Printed On: 4/1/19 9:38:00 AM
As of Last Complete Printing
Number of Pages: 9
Number of Words: 823
Number of Characters: 4,940 (approx.)
